# The Role of Dual Antiplatelet Therapy (DAPT) vs Surgery in a Case of Moyamoya Disease: A Case Report and Review of the Literature

**DOI:** 10.7759/cureus.39694

**Published:** 2023-05-30

**Authors:** Ruchi Yadav, Sindhu C Pokhriyal, Vivek Yadav, Iyad Idries, Ketevan Berekashvili, Kalpana Panigrahi, Mustafa Wasifuddin

**Affiliations:** 1 Hematology and Oncology, Brookdale University Hospital Medical Center, New York, USA; 2 Internal Medicine, One Brooklyn Health, New York, USA; 3 Pulmonary and Critical Care, State University of New York Downstate Health Sciences University, New York, USA; 4 Internal Medicine, Brookdale University Hospital Medical Center, New York, USA; 5 Neurology, Brookdale University Hospital Medical Center, New York, USA; 6 Internal Medicine, Interfaith Medical Center, New York, USA

**Keywords:** vascular neurology, neuro-surgery, stroke, moya moya syndrome, moya moya disease

## Abstract

Moyamoya disease (MMD) is a rare cerebrovascular disease characterized by non-atherosclerotic and non-inflammatory progressive narrowing of the intracranial part of the carotid artery and its proximal branches. The disease process is commonly associated with the development of weak, dilated collateral blood vessels at the base of the brain. This gives it a classic smoky appearance on cerebral angiograms and hence the name “Moyamoya” which means “puff of smoke” in Japanese. When a patient has similar vasculopathy in the setting of another disease then it is known as Moyamoya syndrome (MMS). The associated diseases are sickle cell anemia, neurofibromatosis, long-standing diabetes, uncontrolled hypertension, or chemotherapy.

Despite being known as a disease of the East Asian population, the disease is no longer exclusive to Asians, as evidenced by the rising incidence among non-Asian groups such as Caucasians, Hispanics, and African Americans. Patients can remain asymptomatic or present with ischemic or hemorrhagic stroke, headache, seizures, or recurrent transient ischemic attacks. Conventional cerebral angiography is considered the gold standard for diagnosing MMD. Treatment may be supportive, medical, or surgical.

We present the case of a 42-year-old African American woman with several comorbidities who presented with sudden onset of ischemic stroke and upon further workup was found to have MMD. Equally important is to identify the most effective therapeutic approaches based on individual patients to achieve better clinical outcomes. Our case report highlights the importance of surgery in symptomatic MMD with a lack of supporting evidence indicating the benefits of dual antiplatelet therapy (DAPT).

## Introduction

Moyamoya disease (MMD) is a form of chronic progressive cerebrovascular occlusive disorder characterized by endothelial hyperplasia and fibrosis of the internal carotid artery and its branches [1]. The disease is characterized by progressive stenotic or obstructive changes in the internal carotid artery (ICA), middle cerebral artery (MCA), and/or proximal anterior cerebral artery (ACA), along with the formation of collaterals, giving the smoke-like appearance of blood vessels (Moyamoya = puff of smoke) at the base of the skull [2]. First-line diagnostic imaging includes CT angiography (CTA) and MRI angiography (MRA) to identify the formation of collateral vessels as well as digital subtraction angiography (DSA), which continues to be the gold standard for diagnosing MMD [3,4]. The etiopathogenesis remains unclear, however, several angiogenesis-related factors such as endothelial colony forming cells, vascular endothelial growth factor, transforming growth factor beta 1, and basic fibroblast growth factor are implicated, with genetic polymorphism playing an important role [3].

MMD can occur in children as well as in adults with a bimodal distribution of age showing a peak incidence between 5-9 years and then again between 35-39 years of age [5]. The term MMD was first coined by Suzuki and Takaku in 1969, and the first description of the pathological manifestations of MMD was made in 1957 by Takeuchi and Shimizu for bilateral hypoplasia of the ICA [6]. The incidence of MMD depicts regional differences with a higher incidence in Eastern countries - Japan 0.94/100,000, South Korea 2.3/100,000 - and a lower incidence in North America 0.09/100,000, but exhibiting an upward trend in the U.S. [7]. Patients may present with symptoms such as headache, seizures, facial or limb weakness, and dysarthria [3]. Cerebral ischemia and cerebral hemorrhage are the two major presenting symptoms of MMD with about half of all adult patients presenting with intracranial hemorrhage, and the other half with ischemic symptoms [8]. Treatment may be conservative, medical, or surgical with a revascularizing procedure being considered as definitive leading to restoration of blood flow to the brain by opening the constricted blood vessels or bypassing the obstructed arteries [9]. MMD is a progressive neurological disease associated with new ischemic or hemorrhagic events that are difficult to predict hence our case report emphasizes the importance of timely diagnosis and prompt surgical intervention to help prevent any catastrophic future events.

## Case presentation

**Figure 1:**
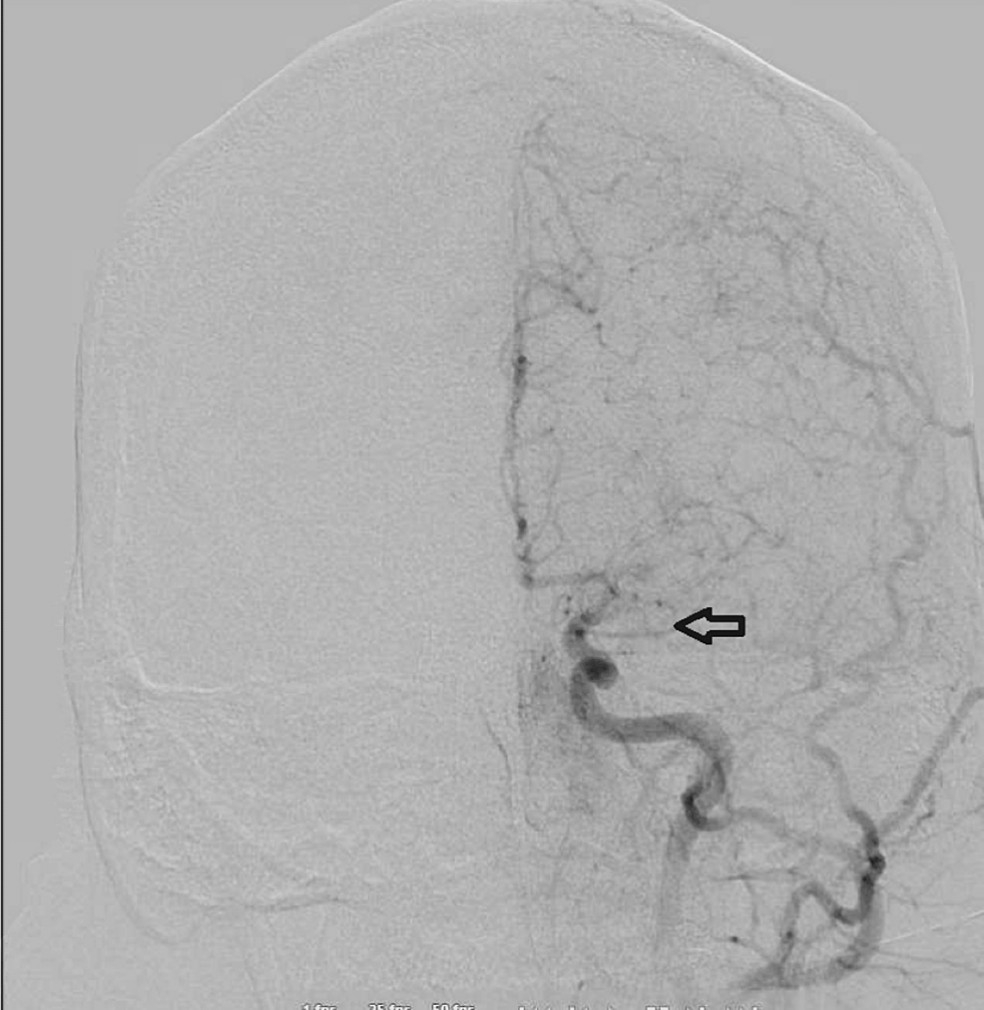
Left common carotid artery (CCA) run anteroposterior view - arrow pointing to abnormal M1

**Figure 2:**
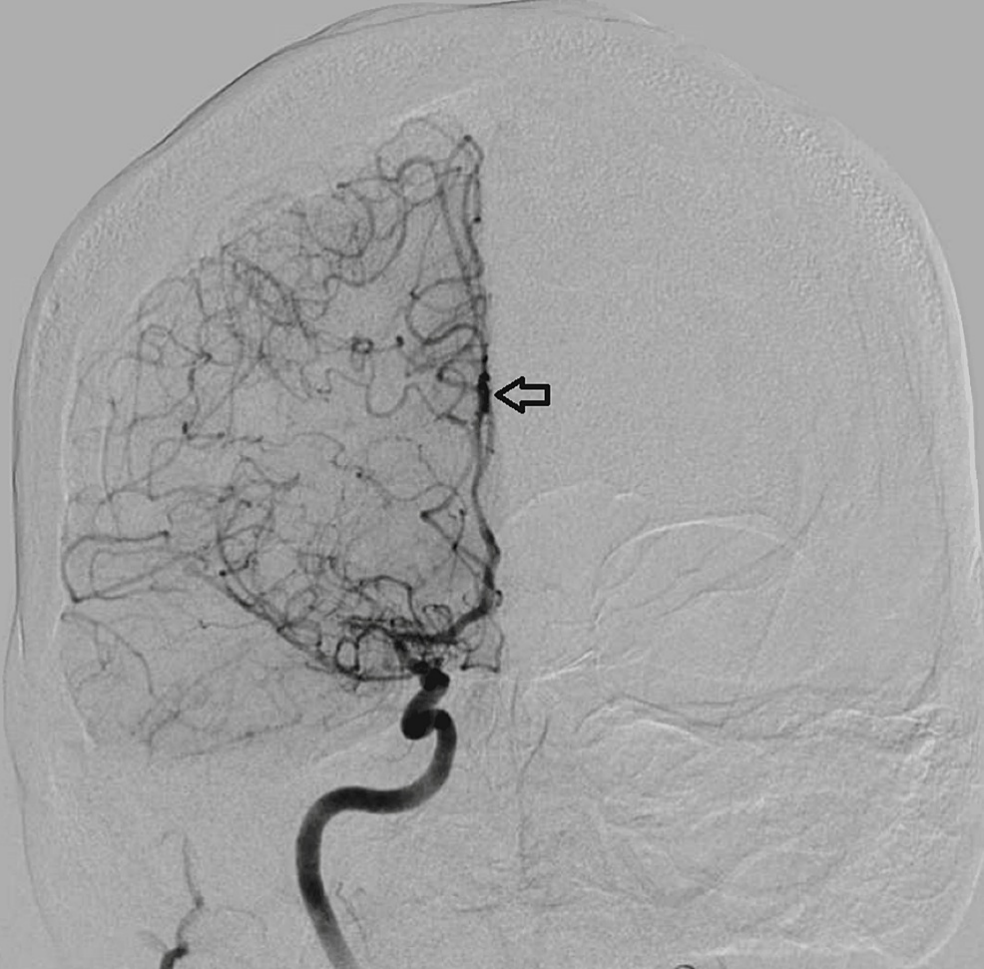
Right internal carotid artery run anteroposterior view - arrow pointing to 'smokey' appearance

**Figure 3:**
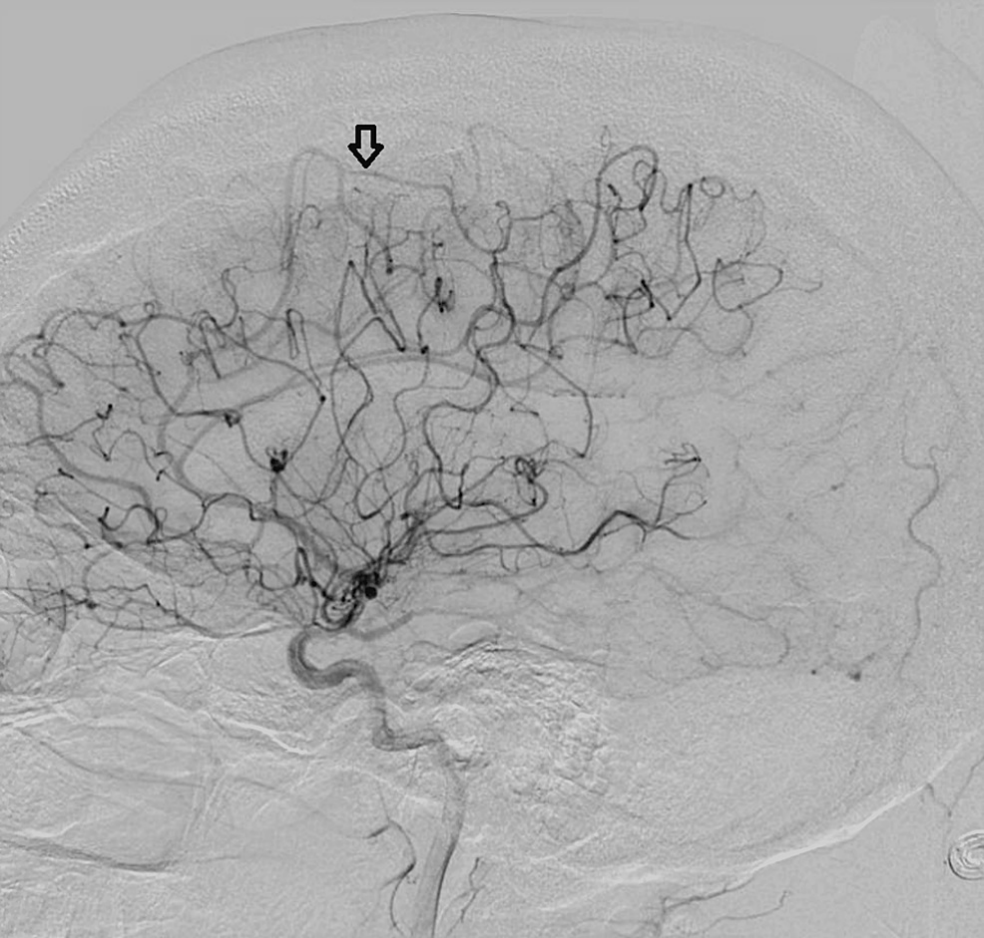
Right internal carotid artery run lateral view late phase - arrow pointing to A3 segment of the right carotid artery

**Figure 4:**
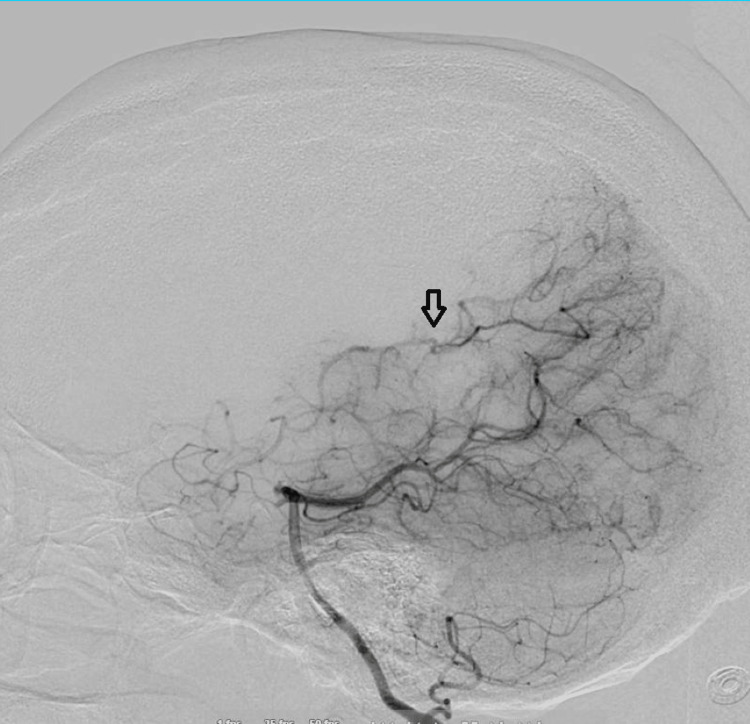
Right vertebral artery lateral projection - arrow pointing to 'smokey' appearance

A 42-year-old African American woman, who is a nurse by occupation, with a past medical history of hypertension, diabetes mellitus, hyperlipidemia, iron deficiency anemia, and morbid obesity presented to the emergency department (ED) with facial weakness and dysarthria which had started 30 minutes before the presentation. On examination, her pulse was 89 beats per min, blood pressure was 96/48 mm Hg, temperature 98.4 °F (oral), respiratory rate 19/min, and Body Mass Index 48.59 kg/m². The cardiovascular, respiratory, and abdominal examinations were unremarkable. On neurological examination, she was alert and oriented to time, place, and person. Glasgow coma scale (GCS) score was 15/15. Pupils were equal at 3 mm and briskly reactive to bright light. The primary gaze was normal and extraocular movements were full. There was a slight delay in the right lip with smile. On the motor exam, there was no drift, and the muscle tone and strength were normal in all four limbs. The sensory exam was normal. Blood work was unremarkable other than hemoglobin of 7.1 gm/dL (reference range 11.4-15.5 g/dL) and hematocrit of 20% (reference range 37-44%). On further work up, iron deficiency anemia was confirmed by ferritin of 9 ng/ml (reference range 11-306 ng/ml), serum iron of 28 μg/ml (11-306 μg/ml), total iron binding capacity of 378 μg/ml (reference range 240-450 μg/ml), transferrin saturation of 270 mg/ml (reference range 206-380 mg/ml). MRI brain done showed an acute left lacunar ischemic stroke (Figure 1). CT angiogram of the head and neck findings showed an abnormality in the right middle cerebral artery (MCA), with the M1 segment of the right MCA difficult to visualize and decreased in caliber. 80% stenosis was seen over a 1 cm long segment involving the proximal M1 segment of the right MCA and 80% stenosis was seen over another 0.5 cm long segment involving the proximal M1 segment of the left MCA (Figures 1-4 show the moyamoya appearance on the CT angiography of the head and neck).

**Figure 5:**
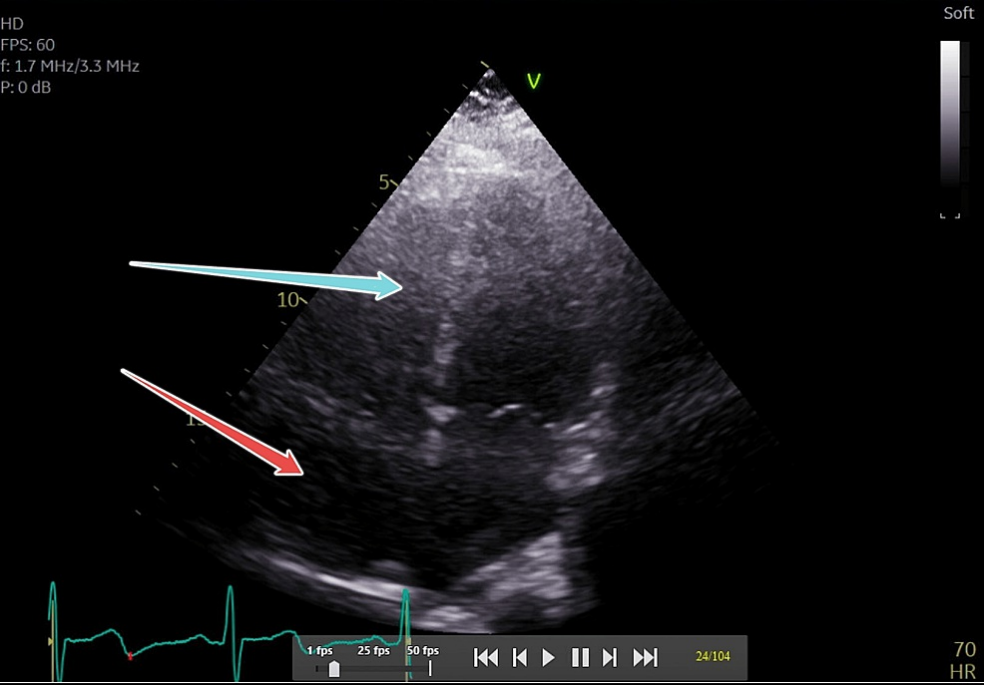
Transthoracic echocardiogram showing mild-left atrial dilation - the red arrow pointing to the right atrium and the blue arrow pointing to the right ventricle for reference

The extracranial arterial structures were normal. These findings were suggestive of left lacunar ischemic stroke with an etiology likely related to Moyamoya vasculopathy. She was offered a tissue plasminogen activator (t-PA) which she declined. She was started on aspirin 81 mg, atorvastatin 80 mg, clopidogrel 75 mg. A transthoracic echocardiogram showed mild left atrial dilatation (Figure 5). The left ventricular ejection fraction was 65%. She continued to have neurological checks every hour for the first 6 hours followed by every 4 hours for the next 24 hours. During her stay in the medical intensive care unit (MICU), she had recurrent symptoms prompting stroke alert but was not treated with IV tPA on either occasion as the patient had declined the treatment. MRI brain repeated three days later showed no new areas of infarction. The deep white matter in the left frontal lobe showed a small evolving ischemic stroke. There was no hemorrhage. There were no FLAIR (fluid attenuated inversion recovery) signal abnormalities except for known periventricular ischemic stroke. The patient was managed medically with supportive measures and was continued on dual antiplatelet therapy (DAPT) and intensive dose statin therapy. Apart from this, she was also on amlodipine 5 mg, telmisartan 40 mg, metoprolol succinate 25 mg, and ferrous sulfate 325 mg. The patient was discharged on the fifth day of admission with a plan to follow up with a tertiary care facility for any needed necessary neurosurgical intervention for MMD. A week after discharge, the patient underwent a left frontotemporal craniotomy and direct extracranial to intracranial bypass procedure for restoration of blood supply to the affected areas and is recovering well. The aspirin and clopidogrel were discontinued and the dosage of atorvastatin was reduced to 40 mg at bedtime.

## Discussion

MMD is a rare, chronic, progressive, occlusive cerebrovascular disease characterized by the development of a network of collaterals resembling a ‘puff of smoke’ appearance on cerebral angiogram [10]. MMD is a rare disease in the US, with just 0.086 newly diagnosed cases per 100,000 individuals per year amounting to approximately one per million new cases annually [9]. As per a study conducted by Kainth et al the incidence in African Americans is 0.13/100,000 person-years as compared to the reported incidence of 0.35/100,000 person-years in Japan [7]. There has been approximately a four-fold increase in the prevalence of Moyamoya disease in the USA likely as a result of improved disease awareness and diagnosis, but may also represent a growth in the disease prevalence [7].

Previously, diagnostic criteria for MMD required the presence of bilateral disease however as per revised guidelines by the Research Committee of MMD of the Japanese Ministry of Health, Labour, and Welfare in 2021, the diagnosis included unilateral disease [11]. Cerebral angiography is the diagnostic test of choice with the presence of stenosis or occlusion in the arteries centered on the terminal portion of the intracranial ICA and Moyamoya vessels (abnormal vascular networks) in the vicinity of the occlusive or stenotic lesions in the arterial phase [11]. The key modification done was the inclusion of both bilateral and unilateral cases of MMD. With advancements in technology, less invasive tests such as Magnetic Resonance Imaging (MRI) or MRA (Magnetic Resonance Angiography) may also be used to diagnose MMD provided there is the presence of all the following criteria - stenosis or occlusion of the terminal portion of the intracranial ICA, decrease in the outer diameter of the terminal portion of the ICA and the horizontal portion of the MCA bilaterally, and abnormal vascular networks in the basal ganglia and/or periventricular white matter on MRA [11]. To differentiate atherosclerotic lesions from MMD, it is imperative to confirm the presence of a decrease in the outer diameter of the involved arteries on heavy T2-weighted MRI [11].

MMD is primarily associated with cerebral ischemic events because of the progressive narrowing of cerebral vessels leading to hypoperfusion to the areas of the brain supplied by them. Frequent recurrent transient ischemic attacks (TIA) are the most common presentation at 37% [12]. Other manifestations include ischemic strokes (17%), hemorrhagic strokes (19%), headaches (6%), epilepsy (3%), and asymptomatic (3%) [13]. Interestingly, hemorrhagic strokes were noted to be lower than ischemic strokes in a large study at a university hospital in the US at 13% [14]. Our patient was presented with an ischemic stroke initially and had recurrent cerebrovascular incidents during her admission to the hospital which aligns with the recurrent nature of the clinical manifestations of MMD.

The cause of MMD has remained elusive for decades. The complicated pathologic features of the stenotic segments of MMD and the unknown nature of the neo-vascularization suggest that MMD pathophysiology is a complex process. Due to the disease’s increased incidence among patients of Asian descent, genetic factors were considered to play a role in its pathogenesis with recent genetic studies demonstrating the ring finger protein 213 gene (RNF213) as an important MMD susceptibility gene among patients of East Asian countries [15]. Lately, the role of immune reactivity along with the presence of vascular endothelial growth factor (VEGF), fibroblast growth factor (FGF), transforming growth factor (TGF-b1), and other cytokines have been implicated in causing vessel narrowing with compensatory vessel hyperplasia [16]. Our patient falls in the spectrum of the African American female population in the USA, with 18-44 years of age distribution, with a complicated recurrent clinical event in the setting of initial treatment with DAPT followed by direct bypass surgery. Understanding the socioeconomic and demographic factors (i.e., age, sex, race/ethnicity) involved in MMD will provide better direction to researchers attempting to elucidate the etiology or address healthcare inequalities.

Currently, no evidence is available suggesting that medical treatment can delay or even reverse the progression of MMD. The available drug treatment only targets its clinical symptoms, including ischemia and hemorrhage, by exerting anti-coagulant or hemostatic effects. Anti-platelet aggregation drugs, as advised by the Japanese guidelines from 2012, are used for the treatment of ischemic MMD but carry the potential risk of bleeding [13]. The rationale for using DAPT is the prevention of thrombogenesis. However, the pathology of ischemic events in MMD may not necessarily have thrombotic etiology hence the controversial role of DAPT [17]. Antiplatelet and antithrombotic agents can be used as an alternative in the settings of surgical contraindications and for patients before revascularization. Our patient initially refused the surgical treatment offered to her hence was placed on DAPT which was later discontinued after the direct bypass procedure.

The treatment of MMD is primarily surgical. Direct surgical techniques involve bypass surgery involving various branches of the carotids including the creation of an external to internal carotid artery bypass [18]. Our patient underwent a left frontotemporal craniotomy and direct extracranial to intracranial bypass. Indirect revascularization involves a variety of techniques mainly including encephalomyosynangiosis, multiple burr hole surgery, encephaloduroarteriosynangiosis, encephaloduromyoarteriosynangiosis, encephaloduromyoarteriopericranial synangiosis, and omental transplantation [19]. Surgery remains an important treatment for MMD, but an individualized treatment strategy should be selected based on risk factors, genetic makeup, clinical presentation, and social structure of each patient.

## Conclusions

MMD represents a heterogeneous syndrome rather than a single disease with an overall rare incidence in the USA but showing an upward trend in prevalence in the last couple of decades. Early diagnosis and initial management are crucial to improve the long-term outcomes of patients. Surgery remains a definitive treatment for ischemic MMD with an overall good prognosis. Medical therapy for symptomatic MMD includes anti-lipid drugs such as statins with no role of DAPT. We need further epidemiological and follow-up studies to understand the natural course of symptomatic MMD with clearly defined roles of medical and surgical management.
